# Clinical features and outcomes of elderly hospitalised patients with chronic obstructive pulmonary disease, heart failure or both

**DOI:** 10.1007/s11739-023-03207-w

**Published:** 2023-02-11

**Authors:** Ernesto Crisafulli, Giulia Sartori, Alice Vianello, Fabiana Busti, Alessandro Nobili, Pier Mannuccio Mannucci, Domenico Girelli, REPOSI Investigators, Pier Mannuccio Mannucci, Giorgio Sesti, Antonello Pietrangelo, Francesco Perticone, Francesco Violi, Salvatore Corrao, Alessandra Marengoni, Mauro Tettamanti, Luca Pasina, Carlotta Franchi, Pier Mannuccio Mannucci, Alessandro Nobili, Giorgio Sesti, Antonello Pietrangelo, Francesco Perticone, Francesco Violi, Salvatore Corrao, Alessandra Marengoni, Mauro Tettamanti, Luca Pasina, Carlotta Franchi, Carlotta Franchi, Alessio Novella, Mauro Tettamanti, Gabriella Miglio, Mauro Tettamanti, Alessia Antonella Galbussera, Ilaria Ardoino, Alessio Novella, Domenico Prisco, Elena Silvestri, Giacomo Emmi, Alessandra Bettiol, Irene Mattioli, Gianni Biolo, Michela Zanetti, Giacomo Bartelloni, Michele Zaccari, Massimiliano Chiuch, Massimo Vanoli, Giulia Grignani, Edoardo Alessandro Pulixi, Matteo Pirro, Graziana Lupattelli, Vanessa Bianconi, Riccardo Alcidi, Alessia Giotta, Massimo R. Mannarino, Domenico Girelli, Fabiana Busti, Giacomo Marchi, Mario Barbagallo, Ligia Dominguez, Vincenza Beneduce, Federica Cacioppo, Salvatore Corrao, Giuseppe Natoli, Salvatore Mularo, Massimo Raspanti, Christiano Argano, Federica Cavallaro, Marco Zoli, Maria Laura Matacena, Giuseppe Orio, Eleonora Magnolfi, Giovanni Serafini, Angelo Simili, Mattia Brunori, Ilaria Lazzari, Angelo Simili, Maria Domenica Cappellini, Giovanna Fabio, Margherita Migone De Amicis, Giacomo De Luca, Natalia Scaramellini, Valeria Di Stefano, Simona Leoni, Sonia Seghezzi, Alessandra Danuto Di Mauro, Diletta Maira, Marta Mancarella, Tiziano Lucchi, Paolo Dionigi Rossi, Marta Clerici, Simona Leoni, Alessandra Danuta Di Mauro, Giulia Bonini, Federica Conti, Silvia Prolo, Maddalena Fabrizi, Miriana Martelengo, Giulia Vigani, Paola Nicolini, Antonio Di Sabatino, Emanuela Miceli, Marco VincenzoLenti, Martina Pisati, Lavinia Pitotti, Donatella Padula, Valentina Antoci, Ginevra Cambiè, Roberto Pontremoli, Valentina Beccati, Giulia Nobili, Giovanna Leoncini, Jacopo Alberto, Federico Cattaneo, Luigi Anastasio, Lucia Sofia, Maria Carbone, Francesco Cipollone, Maria Teresa Guagnano, Ilaria Rossi, Emanuele Valeriani, Damiani D’Ardes, Lucia Esposito, Simona Sestili, Ermanno Angelucci, Gerardo Mancuso, Daniela Calipari, Mosè Bartone, Giuseppe Delitala, Maria Berria, Alessandro Delitala, Maurizio Muscaritoli, Alessio Molfino, Enrico Petrillo, Antonella Giorgi, Christian Gracin, Giovanni Imbimbo, Giuseppe Zuccalà, Gabriella D’Aurizio, Giuseppe Romanelli, Alessandra Marengoni, Andrea Volpini, Daniela Lucente, Francesca Manzoni, Annalisa Pirozzi, Alberto Zucchelli, Antonio Picardi, Umberto Vespasiani Gentilucci, Paolo Gallo, Chiara Dell’Unto, Giuseppe Bellelli, Maurizio Corsi, Cesare Antonucci, Chiara Sidoli, Giulia Principato, Alessandra Bonfanti, Hajnalka Szabo, Paolo Mazzola, Andrea Piazzoli, Maurizio Corsi, Franco Arturi, Elena Succurro, Bruno Tassone, Federica Giofrè, Maria Grazia Serra, Maria Antonietta Bleve, Antonio Brucato, Teresa De Falco, Enrica Negro, Martino Brenna, Lucia Trotta, Giovanni Lorenzo Squintani, Maria Luisa Randi, Fabrizio Fabris, Irene Bertozzi, Giulia Bogoni, Maria Victoria Rabuini, Tancredi Prandini, Francesco Ratti, Chiara Zurlo, Lorenzo Cerruti, Elisabetta Cosi, Roberto Manfredini, Benedetta Boari, Alfredo Giorgi, Ruana Tiseo, Giulia Marta Viglione, Caterina Savriè, Giuseppe Paolisso, Maria Rosaria Rizzo, Claudia Catalano, Irene Di Meo, Claudio Borghi, Enrico Strocchi, Eugenia Ianniello, Mario Soldati, Silvia Schiavone, Alessio Bragagni, Francesca Giulia Leoni, Valeria Sando, Sara Scarduelli, Michela Cammarosano, Ilenia Pareo, Carlo Sabbà, Patrizia Suppressa, Giovanni Michele De Vincenzo, Alessio Comitangelo, Emanuele Amoruso, Carlo Custodero, Giuseppe Re, Andrea Schilardi, Francesca Loparco, Luigi Fenoglio, Andrea Falcetta, Alessia Valentina Giraudo, Salvatore D’Aniano, Anna L Fracanzani, Silvia Tiraboschi, Annalisa Cespiati, Giovanna Oberti, Giordano Sigon, Felice Cinque, Flora Peyvandi, Raffaella Rossio, Giulia Colombo, Pasquale Agosti, Erica Pagliaro, Eleonora Semproni, Canetta Ciro, Valter Monzani, Valeria Savojardo, Giuliana Ceriani, Christian Folli, Francesco Salerno, Giada Pallini, Fabrizio Montecucco, Luciano Ottonello, Lara Caserza, Giulia Vischi, Salam Kassem, Luca Liberale, Nicola Lucio Liberato, Tiziana Tognin, Francesco Purrello, Antonino Di Pino, Salvatore Piro, Renzo Rozzini, Lina Falanga, Maria Stella Pisciotta, Francesco Baffa Bellucci, Stefano Buffelli, Camillo Ferrandina, Francesca Mazzeo, Elena Spazzini, Giulia Cono, Giulia Cesaroni, Giuseppe Montrucchio, Paolo Peasso, Edoardo Favale, Cesare Poletto, Carl Margaria, Maura Sanino, Francesco Violi, Ludovica Perri, Luigina Guasti, Francesca Rotunno, Luana Castiglioni, Andrea Maresca, Alessandro Squizzato, Leonardo Campiotti, Alessandra Grossi, Roberto Davide Diprizio, Francesco Dentali, Marco Bertolotti, Chiara Mussi, Giulia Lancellotti, Maria Vittoria Libbra, Matteo Galassi, Yasmine Grassi, Alessio Greco, Elena Bigi, Elisa Pellegrini, Laura Orlandi, Giulia Dondi, Lucia Carulli, Angela Sciacqua, Maria Perticone, Rosa Battaglia, Raffaele Maio, Aleandra Scozzafava, Valentino Condoleo, Tania Falbo, Lidia Colangelo, Marco Filice, Elvira Clausi, Vincenzo Stanghellini, Eugenio Ruggeri, Sara del Vecchio, Ilaria Benzoni, Andrea Salvi, Roberto Leonardi, Giampaolo Damiani, Gianluca Moroncini, William Capeci, Massimo Mattioli, Giuseppe Pio Martino, Lorenzo Biondi, Pietro Pettinari, Monica Ormas, Emanuele Filippini, Devis Benfaremo, Roberto Romiti, Riccardo Ghio, Anna Dal Col, Salvatore Minisola, Luciano Colangelo, Mirella Cilli, Giancarlo Labbadia, Antonella Afeltra, Benedetta Marigliano, Maria Elena Pipita, Pietro Castellino, Luca Zanoli, Alfio Gennaro, Agostino Gaudio, Samuele Pignataro, Francesca Mete, Miriam Gino, Guido Moreo, Silvia Prolo, Gloria Pina, Alberto Ballestrero, Fabio Ferrando, Roberta Gonella, Domenico Cerminara, Paolo Setti, Chiara Traversa, Camilla Scarsi, Bruno Graziella, Stefano Baldassarre, Salvatore Fragapani, Gabriella Gruden, Franco Berti, Giuseppe Famularo, Patrizia Tarsitani, Roberto Castello, Michela Pasino, Marcello Giuseppe Maggio, Gian PaoloCeda, Simonetta Morganti, Andrea Artoni, Margherita Grossi, Stefano Del Giacco, Davide Firinu, Giulia Costanzo, Giacomo Argiolas, Giovanni Paoletti, Francesca Losa, Giuseppe Montalto, Anna Licata, Filippo Alessandro Montalto, Angelo Rizzo, Francesco Corica, Giorgio Basile, Antonino Catalano, Federica Bellone, Concetto Principato, Lorenzo Malatino, Benedetta Stancanelli, Valentina Terranova, Salvatore Di Marca, Rosario Di Quattro, Lara La Malfa, Rossella Caruso, Patrizia Mecocci, Carmelinda Ruggiero, Virginia Boccardi, Tiziana Meschi, Andrea Ticinesi, Antonio Nouvenne, Pietro Minuz, Luigi Fondrieschi, Giandomenico NigroImperiale, Sarah Morellini, Mario Pirisi, Gian Paolo Fra, Daniele Sola, Mattia Bellan, Roberto Quadri, Erica Larovere, Marco Novelli, Emilio Simeone, Rosa Scurti, Fabio Tolloso, Roberto Tarquini, Alice Valoriani, Silvia Dolenti, Giulia Vannini, Riccardo Volpi, Pietro Bocchi, Alessandro Vignali, Sergio Harari, Chiara Lonati, Federico Napoli, Italia Aiello, Francesco Purrello, Antonino Di Pino, Teresa Salvatore, Lucio Monaco, Carmen Ricozzi, Alberto Pilotto, Ilaria Indiano, Federica Gandolfo, Franco Laghi Pasini, Pier LeopoldoCapecchi, Ranuccio Nuti, Roberto Valenti, Martina Ruvio, Silvia Cappelli, Alberto Palazzuoli, Mauro Bernardi, Silvia Li Bassi, Luca Santi, Giacomo Zaccherini, Vittorio Durante, Daniela Tirotta, Giovanna Eusebi, Marco Cattaneo, Maria Valentina Amoruso, Paola Fracasso, Cristina Fasolino, Moreno Tresoldi, Enrica Bozzolo, Sarah Damanti, Massimo Porta, Miriam Gino

**Affiliations:** 1 Respiratory Medicine Unit and Section of Internal Medicine, Department of Medicine, University of Verona and Azienda Ospedaliera Universitaria Integrata of Verona, Largo L. A. Scuro, 10, 37124 Verona, Italy; 2 Department of Medicine, Section of Internal Medicine, University of Verona and Azienda Ospedaliera Universitaria Integrata of Verona, Verona, Italy; 3 Department of Health Policy, Institute for Pharmacological Research Mario Negri IRCCS, Milan, Italy; 4 Fondazione IRCCS Ca’ Granda Ospedale Maggiore Policlinico, Angelo Bianchi Bonomi Hemophilia and Thrombosis Center, Milan, Italy

**Keywords:** Chronic obstructive pulmonary disease, Heart failure, Multimorbidity, Mortality, Prognosis, Hospital cure

## Abstract

**Background and objective:**

Chronic obstructive pulmonary disease (COPD) and heart failure (HF) mutually increase the risk of being present in the same patient, especially if older. Whether or not this coexistence may be associated with a worse prognosis is debated. Therefore, employing data derived from the REPOSI register, we evaluated the clinical features and outcomes in a population of elderly patients admitted to internal medicine wards and having COPD, HF or COPD + HF.

**Methods:**

We measured socio-demographic and anthropometric characteristics, severity and prevalence of comorbidities, clinical and laboratory features during hospitalization, mood disorders, functional independence, drug prescriptions and discharge destination. The primary study outcome was the risk of death.

**Results:**

We considered 2,343 elderly hospitalized patients (median age 81 years), of whom 1,154 (49%) had COPD, 813 (35%) HF, and 376 (16%) COPD + HF. Patients with COPD + HF had different characteristics than those with COPD or HF, such as a higher prevalence of previous hospitalizations, comorbidities (especially chronic kidney disease), higher respiratory rate at admission and number of prescribed drugs. Patients with COPD + HF (hazard ratio HR 1.74, 95% confidence intervals CI 1.16–2.61) and patients with dementia (HR 1.75, 95% CI 1.06–2.90) had a higher risk of death at one year. The Kaplan–Meier curves showed a higher mortality risk in the group of patients with COPD + HF for all causes (*p* = 0.010), respiratory causes (*p* = 0.006), cardiovascular causes (*p* = 0.046) and respiratory plus cardiovascular causes (*p* = 0.009).

**Conclusion:**

In this real-life cohort of hospitalized elderly patients, the coexistence of COPD and HF significantly worsened prognosis at one year. This finding may help to better define the care needs of this population.

## Introduction

Chronic obstructive pulmonary disease (COPD) and heart failure (HF) are prevalent clinical conditions among older patients [1], frequently coexisting in the same multimorbid individual [2]. Compared with a general population, patients with COPD are more than twofold likely to have a cardiovascular disease [3], while approximately 1 in 7 patients with heart failure, even those with preserved ejection fraction, have concomitant COPD [4]. Smoking habits and chronic systemic inflammation are commonly shared risk and mechanistic factors [5], and advanced age may increase the prevalence of this association. In a real-life setting of internal medicine and geriatric wards and in a cohort of elderly patients with multimorbidity, inpatients with COPD had a higher prevalence of HF (29%) than those without (18%) [6].

Although management and treatment differ, breathlessness may make it difficult to diagnose and distinguish COPD from HF [7]. Studies comparing these two diseases for some clinical outcomes focus on exciting considerations on similarities and differences**,** but findings often disagree. Taken separately, COPD and HF had a similar risk of hospitalization and death at three years in an outpatient setting [8]. However, when COPD and HF occur together, their coexistence may be associated with a worse outcome than either condition alone [9–11], suggesting a dangerous interaction between the two diseases [12], even in the context of a very long follow-up of community-dwelling elderly subjects [13]. Such an additive prognostic effect seems to vary in the literature according to the selection criteria employed for the different index patient populations, being slightly more evident in COPD with HF [9, 14] than in HF with COPD [15]. In the latter category of outpatients, the coexistence of other comorbidities (such as chronic kidney disease, anemia or diabetes) makes this association less strong [15]. Similarly, data from a long-term registry of the European Society of Cardiology Heart Failure show that in hospitalized HF patients, the concomitant presence of COPD did not increase significantly the risk of all-cause mortality at one-year [16]. On the other hand, in a recent meta-analysis including 18 studies (6 post-hoc analyses of randomized controlled trials and 12 observational studies), COPD, if present in HF patients was associated with a 24% increased risk for all-cause mortality but not for cardiovascular mortality [17]. Thus, further evidence is needed concerning the role that COPD and HF may have in the same patient.

The association between COPD and HF has been generally evaluated in outpatients and hospitalized patients in the context of specialized care settings, pneumological or cardiological. Such a two-cohort approach (e.g., starting from patients with a well-known/prevalent disease with or without another less-known/secondary disease) allows to observe only an added effect to the original cohort [9, 15–17]. However, less information is available in other settings, particularly in the real life of internal medicine wards, characterized by a holistic approach to hospitalized elderly patients and multiple chronic conditions, thus allowing an accurate evaluation of the relative weight of each condition. With this background and gaps of knowledge, our working hypothesis was that only the coexistence of both diseases in the same patient would influence clinical outcome. Therefore, we chose to describe clinical features, hospital care and outcomes of elderly inpatients with COPD, HF or both admitted to internal medicine and geriatric hospital wards participating in the REPOSI registry.

## Methods

### Study population

This retrospective cross-sectional study analyzed data from the REPOSI registry in the recruitment years spanning from 2010 to 2018. REPOSI, an independent registry run by the Italian Society of Internal Medicine (SIMI), the Mario Negri Institute for Pharmacological Research and the IRCCS Foundation Maggiore Policlinico Hospital, involved a network of internal medicine and geriatric wards in order to collect data on polypharmacy in elderly patients often affected by multiple diseases [18]. The registry design is accessible on the related website [19]. Patients 65 years or older who gave informed consent and were admitted to Internal or Geriatric Medicine wards during the four index weeks chosen for recruitment each year (in February, June, September and December) were eligible for REPOSI. During each index week, data concerning socio-demographic details, the main reason for admission and comorbidities, diagnoses, drug treatment, clinical events during hospitalization and outcome were recorded in at least ten consecutively enrolled patients from each ward. In addition, data on mortality or any new hospitalization were collected by a telephone interview performed by a physician three and twelve months after hospital discharge.

Subjects were referred to as having COPD or HF if a disease diagnosis was reported in medical charts or the diagnosis was made at hospital admission, according to the codes reported in the International Classification of Diseases system (ICD-9), 9th Edition. We considered the ICD-9 codes 491.x, 492.x, and all subsequent subcodes to define patients with COPD and the ICD-9 code 428.x and all subsequent subcodes to define patients with HF. Patients with both conditions were considered as a separate group (COPD + HF). Other comorbidities related to specific ICD-9 codes were reported (see Appendix).

### Measurements and outcomes

In the three diagnosis groups (COPD, HF, COPD + HF), we collected socio-demographic and anthropometric variables such as age, sex, body mass index (BMI), marital status, living arrangement, low-income work, years of education and need for a caregiver in the activities of daily living. Moreover, variables concerning a previous institutionalization or hospitalization, smoking and alcohol habits’, severity and comorbidity impact as assessed by the Cumulative Illness Rating Scale-CIRS [20, 21] and the prevalence of the more common diseases (hypertension, hypercholesterolemia, coronary artery disease, atrial fibrillation, peripheral arterial disease, diabetes, chronic kidney disease, osteoporosis, dementia, depression, cancer) were also collected.

On admission, we evaluated the following characteristics: body temperature, systolic and diastolic blood pressure, heart and respiratory rate, laboratory data (fasting glucose, creatinine, hemoglobin, erythrocytes, mean corpuscular volume, leukocytes, platelets, cholesterol, albumin, prothrombin time), oxygen saturation by pulse oximetry (SpO_2_), presence of pressure ulcers or need for a urinary catheter, cognitive status and mood disorders (by the Short Blessed Test-SBT [22] and the Geriatric Depression Scale-GDS [23], respectively). Furthermore, functional independence (by the Barthel Index-BI [24]), drug prescriptions (at admission, during hospitalization, at three months and one-year follow-up) and the place of destination at discharge were also evaluated.

The primary outcome was the risk of death, evaluated during hospitalization and follow-ups at three, six, and twelve months. The risk of death has been considered for all causes, respiratory causes only, cardiovascular causes only and respiratory and cardiovascular causes together. Other outcomes were duration of hospital stay and readmission rate (at three months and one-year follow-up).

### Statistical analysis

A preliminary Shapiro–Wilk test was performed. Data having a non-normal distribution have been reported as numbers (percentages) for categorical variables and medians [1st quartile; 3rd quartile] for continuous variables. Categorical variables were compared using the chi square test or the Freeman-Halton extension of Fisher’s exact test [25], while continuous variables were assessed by the non-parametric Kruskal–Wallis H or Mann–Whitney U tests, as appropriate.

Cox proportional hazard regression models were used to predict the risk of death for all causes [26]. The hazard ratio (HR) and 95% confidence intervals (CI) were calculated. Time-to-event variables were analyzed using Kaplan–Meier survival curves; the Gehan–Breslow–Wilcoxon test was applied due to its ability to emphasize early between-group differences [27].

All analyses were performed using IBM SPSS, version 25.0 (IBM Corp., Armonk, NY, USA) and a *p*-value of < 0.05 has been considered statistically significant.

## Results

Our study population considered 2,343 hospitalized patients, of whom 1,154 (49%) were those with COPD, 813 (35%) with HF and 376 (16%) with COPD + HF. The general characteristics of the study population are reported in Table 1. Differences were found among the three study groups: as compared to COPD patients, HF patients were older, with more females, had more frequent previous hospitalisations and such comorbidities as arterial hypertension, atrial fibrillation, diabetes and chronic kidney disease, as well as the need of a caregiver. HF patients had lower smoking and alcohol habits than COPD patients. On the other hand, patients with COPD + HF (in comparison to COPD alone) were older with more comorbidities (atrial fibrillation, diabetes and chronic kidney disease); while in comparison to HF, they were more frequently males with a higher prevalence of current and former smoking status and alcohol habits. Moreover, patients with COPD + HF had more previous hospitalisations and comorbidities (as assessed by CIRS) than COPD or HF.

**Table 1:** General characteristics of the study population

Variables	COPD (*N* = 1,154)	HF (*N* = 813)	COPD + HF (*N* = 376)	*p*-Value
Age, years (*N* = 2,343)	80 [74; 85]	82 [77; 87] **	82 [76; 87] **	** < 0.001**
Sex, male (*N* = 2,343)	713 (62)	350 (43) **	230 (61) §§	** < 0.001**
BMI, kg∙m^2^ (*N* = 2,037)	25.5 [22.7; 28.7]	25.9 [22.8; 29.4]	26 [23; 29.6]	0.108
Regions of enrollment (*N* = 2,324)				0.965
Northern Italy	616 (54)	430 (54)	198 (53)	
Center Italy	215 (19)	156 (19)	68 (18)	
Southern Italy	316 (27)	217 (27)	108 (29)	
Marital status, married or widow (*N* = 2,251)	1007 (90)	708 (91)	325 (91)	0.737
Living arrangement, alone/partner/sons (*N* = 2,229)	257 (23)/528 (48)/145 (13)	168 (22)/330 (43)/153 (20) *	84 (23)/148 (41)/64 (18)	**0.006**
Low-income work (*N* = 1,714)	672 (80)	504 (84)	234 (86) *	**0.044**
Years of education, mean (95% CI) (*N* = 2,042)#	7.2 (6.9—7.4)	6.9 (6.7—7.3)	6.5 (6.1—6.9) * §	**0.014**
Caregiver (*N* = 2,300)	631 (56)	510 (64) **	234 (63) *	** < 0.001**
Partner/sons/other (*N* = 1,363)	229 (36)/274 (44)/124 (20)	127 (25)/270 (54)/106 (21) **	80 (34)/105 (45)/48 (21) §	**0.001**
Previously institutionalized (*N* = 2,329)	66 (5.8)	55 (6.8)	20 (5.4)	0.534
Previously hospitalized (*N* = 2,056)	428 (43)	362 (51) *	206 (61) ** §	** < 0.001**
Smoking habit, former/current (*N* = 2,276)	576 (51)/187 (17)	267 (34)/33 (4.2) **	200 (54)/41 (11) * §§	** < 0.001**
Pack/year (*N* = 842)	39 [20; 55]	27.5 [12.37; 44.25] **	37.5 [20; 61.25] §§	** < 0.001**
Alcohol habit, former/current (*N* = 2,249)	173 (16)/406 (36)	96 (12)/216 (28) **	48 (14)/128 (35) §	** < 0.001**
CIRS-SI (*N* = 2,341)	1.69 [1.53; 1.92]	1.69 [1.53; 2]	1.85 [1.69; 2.08] ** §§	** < 0.001**
CIRS-CI (*N* = 2,341)	3 [2; 5]	3 [2; 5]	4 [3; 5] ** §§	** < 0.001**
Arterial hypertension (*N* = 2,342)	907 (79)	688 (85) *	312 (83)	**0.003**
Hypercholesterolemia (*N* = 2,343)	61 (5.3)	42 (5.2)	22 (5.9)	0.883
CAD (*N* = 2,343)	278 (24)	193 (24)	108 (29)	0.142
Atrial fibrillation (*N* = 2,343)	287 (25)	356 (44) **	162 (43) **	** < 0.001**
PAD (*N* = 2,343)	64 (5.5)	32 (3.9)	23 (6.1)	0.168
Diabetes (*N* = 2,343)	312 (27)	263 (32) *	139 (37) **	** < 0.001**
Chronic kidney disease (*N* = 2,343)	268 (23)	295 (36) **	164 (44) **§	** < 0.001**
Osteoporosis (*N* = 2,343)	98 (8.5)	55 (6.8)	22 (5.9)	0.153
Dementia (*N* = 2,343)	90 (7.8)	70 (8.6)	23 (6.1)	0.330
Depression (*N* = 2,343)	83 (7.2)	47 (5.8)	18 (4.8)	0.185
Cancer (*N* = 1,311)	74 (12)	55 (11)	33 (15)	0.259

Data are shown as numbers of patients (percentage) or medians [1st quartile; 3rd quartile]. Percentages are calculated for non-missing data

In bold are significant variables

*COPD* indicates Chronic Obstructive Pulmonary Disease, *HF* heart failure, *BMI* body mass index, *CIRS-SI* and *CIRS-CI* Cumulative Illness Rating Scale, severity and comorbidity index, respectively, *CAD* coronary artery disease, *PAD* peripheral arterial disease

^*^ and **p < 0.05 and *p* < 0.001 *versus* COPD

^§^ and §§ *p* < 0.05 and *p* < 0.001 *versus* HF

^#^ Values of medians [25°–75° percentiles] were 5 [5, 8] in all three groups, not descriptive of differences

Functional, clinical and laboratory variables are illustrated in Table 2. At admission, patients with COPD + HF had lower values of SpO_2_ than those with HF, while the respiratory rate was higher in COPD + HF patients as compared to COPD and HF only. Values of SBT for cognitive status were higher in HF (in comparison to COPD), while values of GDS for mood disorders were higher in HF and COPD + HF compared to COPD only. The measure of functional independence (evaluated by BI, ad admission, during hospitalization and three months post-discharge) was worse in patients with HF only and COPD + HF as compared to COPD. Compared with COPD and HF alone, drug prescriptions (at admission, discharge and three months follow-up) were higher in COPD + HF. The need for a urinary catheter was lower in COPD.

**Table 2:** Functional, clinical and laboratory data

Variables	COPD	HF	COPD + HF	*p*-Value
Body temperature, °C (*N* = 2,196)	36.2 [36; 36.8]	36.1 [36; 36.6] *	36 [36; 36.7]	**0.028**
Systolic blood pressure, mmHg (*N* = 2,332)	130 [120; 140]	130 [110; 140] *	125 [110; 140] **	**0.001**
Diastolic blood pressure, mmHg (*N* = 2,331)	70 [65; 80]	70 [60; 80] *	70 [63.75; 80]	**0.003**
Heart rate, bpm (*N* = 2,319)	80 [70; 90]	80 [70; 90]	80 [70; 90]	0.222
Respiratory rate, bpm (*N* = 1,127)	18 [16; 22]	18 [15; 22] *	20 [16; 24] * §§	** < 0.001**
Fasting glucose, mg/dL (*N* = 2,258)	111 [92; 142]	115 [95; 149]	113.5 [96; 150]	0.053
Creatinine, mg/dL (*N* = 2,321)	1.01 [0.8; 1.4]	1.2 [0.9; 1.78] **	1.3 [0.95; 1.78] **	** < 0.001**
Hemoglobin, mg/dL (*N* = 2,328)	12.1 [10.5; 13.6]	11.4 [9.8; 13] **	12 [10.6; 13.3] §§	** < 0.001**
Erythrocytes, million cells per mcL (*N* = 2,307)	4.18 [3.64; 4.61]	4 [3.49; 4.5] **	4.1 [3.7; 4.6] §	**0.001**
Mean corpuscular volume, fL (*N* = 2,312)	89.9 [85; 94]	89.85 [83; 94]	90 [85; 95]	0.099
Leukocytes, cells per microliter (× 10^3^/μL) (*N* = 2,319)	8.58 [6.5; 11.6]	8.16 [6.25; 10.73]	8.5 [6.4; 11.4]	0.060
Platelets (cells per microliter) (× 10^3^/μL) (*N* = 2,323)	222 [170; 282.5]	214 [164; 282.5]	225 [160; 279.5]	0.616
Cholesterol (mg/dL) (*N* = 1,626)	154 [126; 186]	144 [118; 178] **	152 [123.75; 174]	** < 0.001**
Albumin, gr/dL (*N* = 1,417)	3.4 [3; 3.8]	3.3 [2.97; 3.6] *	3.3 [2.9; 3.8]	**0.019**
Prothrombin time—INR (*N* = 2,144)	1.1 [1; 1.25]	1.2 [1.05; 1.76] **	1.16 [1.02; 1.6] **	** < 0.001**
SpO_2_, % (*N* = 1,795)	95 [92; 97]	96 [94; 97] *	95 [92; 97] §§	** < 0.001**
Pressure ulcers (any stages) (*N* = 1,742)	25 (2.9)	30 (5)	12 (4.4)	0.118
Need for urinary catheter (*N* = 2,284)	322 (29)	375 (47) **	171 (46) **	** < 0.001**
Short Blessed Test score (*N* = 2,010)	8 [2; 14]	9 [4; 16] *	9 [4; 16]	**0.020**
Geriatric Depression Scale score (*N* = 1,841)	1 [0; 2]	1 [1; 2] *	2 [1; 3] *	**0.006**
Barthel index				
At admission (*N* = 1,822)	88 [60.75; 100]	80 [51; 95] **	77.5 [52; 92] **	** < 0.001**
During hospitalization (*N* = 2,180)	84 [52; 98]	75 [39; 93] **	72.5 [39.75; 91.25] **	** < 0.001**
At 3-month follow-up (*N* = 1,162)	86 [62; 98]	82 [52; 97] *	80 [57; 93] *	**0.006**
At 12-month follow-up (*N* = 300)	79 [51; 91]	75 [36.5; 95]	77.5 [55.5; 91.5]	0.961
Drugs prescriptions (number of drugs)				
At admission (*N* = 2,335)	6 [4; 8]	7 [5; 9] **	8 [6; 10] ** §§	** < 0.001**
At discharge (*N* = 2,001)	9 [6; 12]	9 [7; 12] *	11 [7; 14] ** §§	** < 0.001**
At 3-month follow-up (*N* = 1,326)	7 [4; 10]	7 [6; 10] *	8 [6; 11] **	** < 0.001**
At 12-month follow-up (*N* = 361)	6 [3; 9]	7 [4; 9]	9 [6; 12] ** §	** < 0.001**
Length of hospital stay, days (*N* = 2,162)	10 [7; 15]	10 [7; 15]	10 [7.25; 14]	0.836
Destination at discharge (*N* = 1,596)				0.207
Home	734 (92)	480 (88)	233 (92)	
Nursing home	31 (3.9)	36 (6.6)	12 (4.7)	
Rehabilitation	24 (3)	22 (4)	8 (3.1)	
Palliative care	7 (0.9)	8 (1.5)	1 (0.4)	
Readmission at 3 months (*N* = 2,044)	154 (15)	98 (14)	52 (16)	0.645
Readmission at 12 months (*N* = 1,946)	178 (18)	107 (16)	65 (22)	0.098

Data are shown as numbers of patients (percentage) or medians [1st quartile; 3rd quartile]. Percentages are calculated for non-missing data.

In bold are significant variables

*COPD* indicates Chronic Obstructive Pulmonary Disease, *HF* heart failure, *SpO*_*2*_ oxygen saturation by pulse oximetry

^*^ and **p < 0.05 and *p* < 0.001 *versus* COPD

^§^ and §§*p* < 0.05 and *p* < 0.001 *versus* HF

Table 3 reports the univariate and multivariate-adjusted Cox regression models predicting the risk of death for all causes at one year. In the univariate analysis, variables significantly increasing the risk of death were: the presence of COPD + HF (reference: COPD), age ≥ 85 years, male sex, underweight (reference: normal weight), a CIRS-CI value ≥ 3, the presence of coronary artery disease, chronic kidney disease, dementia, cancer, a SBT score ≥ 10, a GDS score > 2, and a BI ≤ 40. The overweight and current smokers (versus former) were demonstrated to have a lower risk of death. The multivariate-adjusted model confirmed a statistically significant and independent risk of worse prognosis for patients with COPD + HF (HR 1.74; 95% CI 1.16–2.61; *p* = 0.008) and those with dementia (HR 1.75; 95% CI 1.06–2.90; p = 0.030).

**Table 3:** Univariate and multivariate-adjusted Cox regression models predicting the risk of death for all causes at one year

	Univariate			Multivariate-adjusted	
	HR	*p*-Value	HR		*p*-Value
COPD	1		1		
HF	1.22 (0.96–1.55)	0.102	1.13 (0.77–1.65)		0.541
COPD + HF	1.54 (1.16–2.03)	**0.003**	1.74 (1.16–2.61)		**0.008**
Age, ≥ 85 years	1.82 (1.47–2.25)	** < 0.001**			
Male	1.36 (1.09–1.69)	**0.005**			
Normal weight	1				
Underweight	1.95 (1.41–2.69)	** < 0.001**			
Overweight	0.72 (0.52–0.99)	**0.045**			
Smoking habit, current (versus former)	0.61 (0.40–0.92)	**0.019**			
CIRS-CI ≥ 3	1.66 (1.29–2.14)	** < 0.001**			
Presence of coronary artery disease	1.27 (1.009–1.60)	**0.042**			
Presence of chronic kidney disease	1.40 (1.12–1.74)	**0.002**			
Presence of dementia	2.18 (1.61–2.95)	** < 0.001**	1.75 (1.06–2.90)		**0.030**
Presence of cancer	1.95 (1.39–2.73)	** < 0.001**			
Overt cognitive impairment (SBT score ≥ 10)	1.91 (1.49–2.45)	** < 0.001**			
Probable depression (GDS score > 2)	1.38 (1.02–1.85)	**0.033**			
Clinically significant disability (BI ≤ 40)	2.66 (2.07–3.41)	** < 0.001**			

The multivariate model has been adjusted for anthropometric variables, smoking habit, comorbidities, overt cognitive impairment, probable depression and clinically significant disability

*COPD* indicates Chronic Obstructive Pulmonary Disease, *HF* heart failure, *HR* hazard ratio, *CIRS-CI* Cumulative Illness Rating Scale, comorbidity index, *SBT* short blessed test, *GDS* geriatric depression scale, *BI* Barthel index

In bold are significant variables

The Hosmer and Lemeshow Test for the multivariate adjusted model was *p* = 0.877

**Fig. 1:**
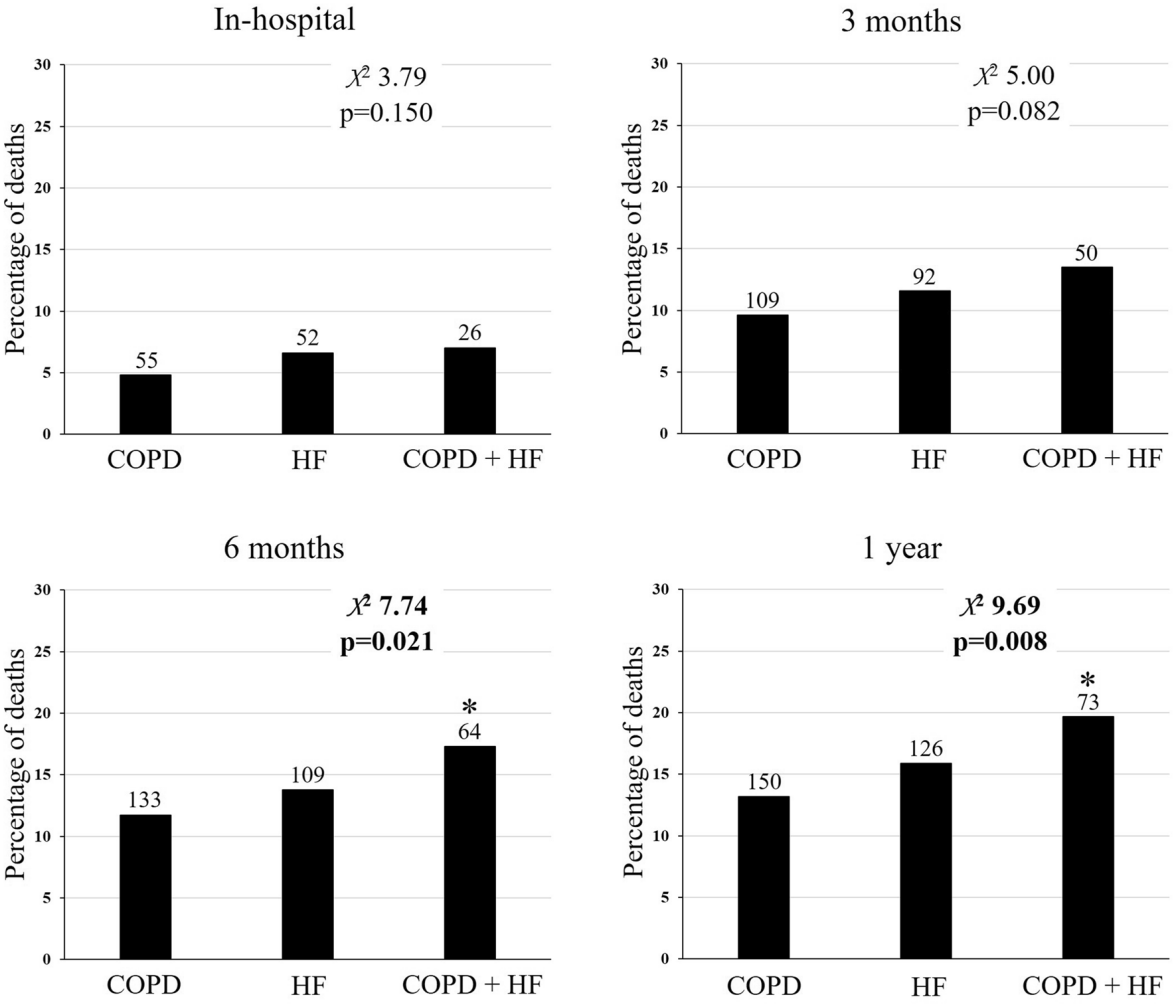
Percentage of deaths during hospitalization and in the follow-up of 3 and 6 months and one year. *p < 0.05 *versus* COPD. Numbers over black bars represent the absolute number of deaths

**Fig. 2:**
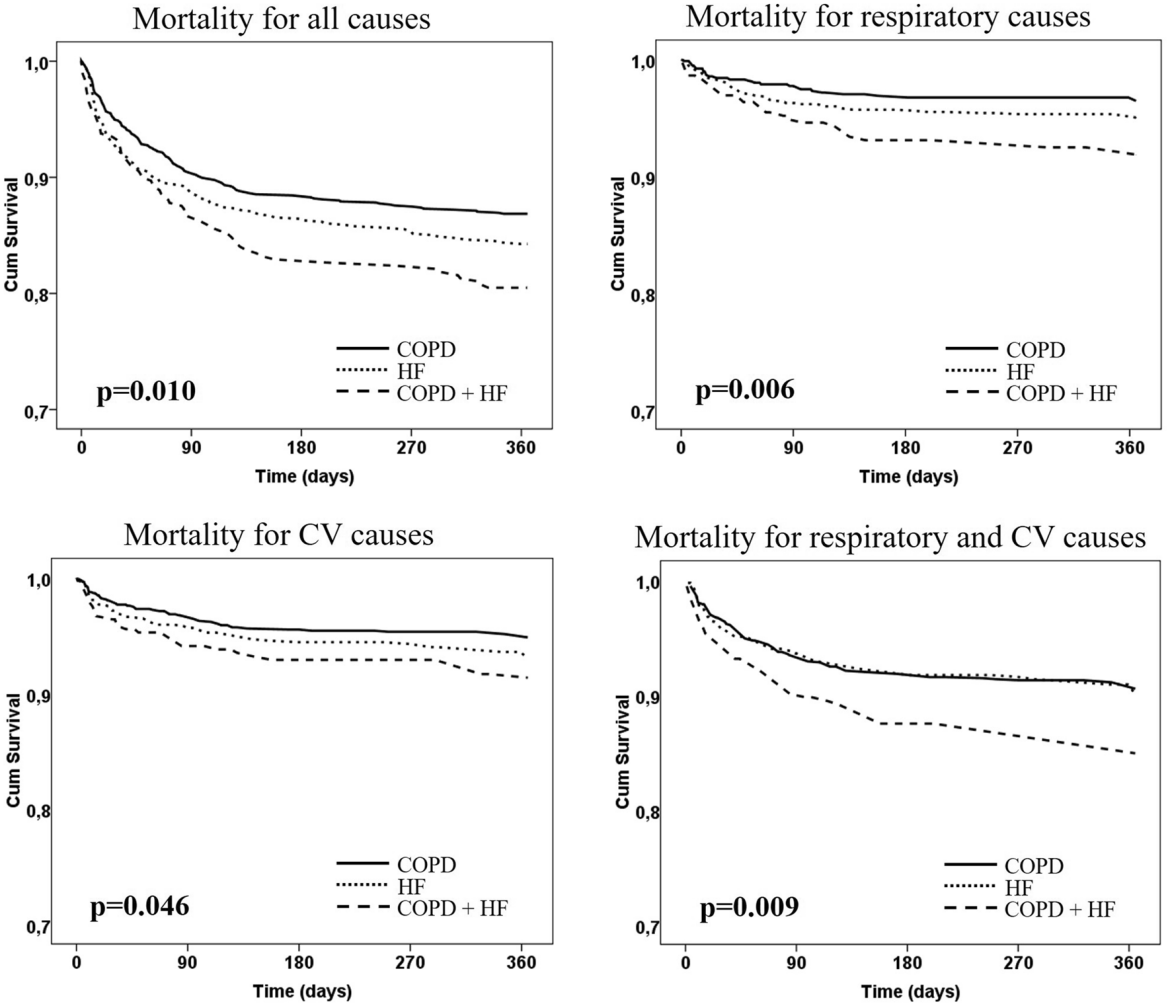
Kaplan–Meier curves. The analysis has been performed with the Gehan–Breslow–Wilcoxon test. *CV* indicates cardiovascular

Figure 1 reported the distribution of survivors/deaths in the three groups of patients. Deaths were significantly more numerous in COPD + HF in the follow-up of six months and one year (*χ*^2^ 7.74, *p* = 0.021 and *χ*^2^ 9.69, *p* = 0.008, respectively). In addition, the Kaplan–Meier curves of the three study groups (Fig. 2) showed a stratified and statistically significant higher risk of mortality for all causes (*p* = 0.010 at Gehan–Breslow–Wilcoxon test), respiratory causes (*p* = 0.006), cardiovascular causes (*p* = 0.046) and respiratory and cardiovascular causes (*p* = 0.009).

## Discussion

An elderly patient with COPD or HF represents a prototype to evaluate chronicity and disease progression, and the internist as a hospitalist is the physician that should typically exercise a holistic approach to complexity [28]. In a large population of elderly patients admitted to internal medicine wards, our findings demonstrate that the coexistence of both diseases in the same patients is associated with clinical peculiarities, particularly a worse prognosis in the context of a follow-up of one year. Therefore, a synergic association may be hypothesized.

The burden of chronicity is globally becoming a major challenge for health care systems. Thus, the identification of the varied clinical characteristics may help to better focus on the health policy administrative-economic efforts, higher health care resource utilization and costs [29], highlighting the possibility for a multidisciplinary and integrated care plan of cure. Our study reinforces these considerations by comparing patients with COPD, HF or COPD + HF. Although some aspects reported herewith are well known, such as the specific phenotypic characteristics of HF patients (prevalently older females in comparison to COPD), we found that the burden of care was progressively higher from COPD to HF, reaching the maximal impact in patients having both the diseases. In them, there were a high number of previous hospitalizations, comorbidities with a peculiar prevalence of chronic kidney disease and also a higher number of drugs prescriptions at admission, discharge and follow-up. All these data identify a clinical subset with a worse mortality prognosis for all causes, evident after 6 months and significant at 1 year (Table 3 and Figures).

Patients with COPD + HF represent clinically complex patients with different phenotypes, in whom baseline variables as sex, age and associated comorbidities have a different pattern of expression. In general, COPD and HF have a higher prevalence in men and women, respectively [30, 31]. However, in the present cohort, patients with both conditions had a sex-specific prevalence similar to COPD alone [32]. Age is a significant risk factor for most chronic diseases and multimorbidity is dramatically present in older patients [33]. The multimorbidity profile of these diseases in the aging process has been studied from a network perspective, revealing that in patients with COPD + HF the number of concomitant chronic conditions is substantially higher than that found in the general population [32]. The burden of comorbidities, shown by selectively higher values of CIRS-SI and CIRS-CI in our patients with COPD + HF, may be of help to direct prevention and management strategies. Of note, the trend in diabetes prevalences (27%, 32% and 37% in COPD, HF, and COPD + HF patients, respectively), along with a higher and significant difference in chronic kidney (44% in patients with COPD + HF) suggests a link among these chronic conditions, all mediated by dysmetabolism and low-grade chronic inflammation [34]. Furthermore, in the last few years HF has been consistently associated with the presence of clonal hemopoiesis of indeterminate potential (CHIP), a common age-related phenomenon characterized by the presence of somatic mutations in clonal leukocytes that are able to drive an aberrant inflammatory response [35]. Recently, also COPD has been associated with CHIP [36], which may therefore be a common ground for COPD and HF that warrants further studies to elucidate the potential pathophysiological link between the two conditions. Nonetheless, the simultaneous presence of both diseases highlights a population of elderly people with a higher morbidity burden, usually associated with a greater risk of polypharmacy [37] and higher use of healthcare services [38, 39]. This aspect is in line with the higher number of drugs prescribed (at admission, discharge and follow-up) (Table 2) and the prevalence of previous hospitalizations (Table 1) in our patients with COPD + HF.

Prognosis in COPD + HF patients has been evaluated in different settings. Although several studies described a worsening effect of COPD and HF when coexisting in the same patient [9, 13, 15–17], data on hospitalized and elderly patients are scarce and do not confirm this finding [16]. However, the methodology of these studies, focused on a reference population (either COPD or HF) in which the additive effect of the other disease was evaluated, is likely to be the reason for discordant results, because these studies were carried out in pulmonological (COPD) or cardiological (HF) subspecialty settings and, thus, with varying diagnostic accuracy and treatment appropriateness [40]. Moreover, they generally considered only two groups (e.g., COPD *vs* COPD + HF or HF *vs* HF + COPD), likely missing the comprehensive multimorbidity context typical of elderly patients as well as the opportunity to observe an interaction effect and the relative weight of both diseases (COPD plus HF) in the same patient. As compared to the existing knowledge, including very recent studies still based on administrative data from outpatients initially selected for a single disease (COPD) [41], the novelty of our study consists into the peculiar internal medicine dataset of elderly hospitalized patients, where we could perform a real-life evaluation by a head-to-head comparison of the three different conditions (COPD, HF and COPD plus HF). This allowed us to evaluate any single difference among them, as well as to distinguish their burden effect on the outcomes. Of note, there were no differences in one-year mortality between COPD and HF alone, while mortality was significantly higher in patients with both diseases. Furthermore, the length of hospital stay was similar in the three groups, confirming that the hospitalization burden was similar. Throughout the entire follow-up period, we noted that although mortality rates during hospitalization and at three months were higher in patients with COPD + HF, this difference became statistically significant (versus COPD) only at six months and one year, perhaps owing to the long-term chronicity and complexity of these patients. Albeit not statistically significant, prognosis seems to be better for COPD alone than for HF alone [9, 15]. In the latter category, older age and a higher prevalence of other comorbidities (atrial fibrillation, diabetes and chronic kidney disease) might explain this result. Finally, in patients with COPD + HF, the higher risk of death was confirmed in the multivariate model adjusted for other common risk factors potentially influencing prognosis, such as anthropometric variables, smoking habit, comorbidities, cognitive impairment, depression and clinically significant disability. This finding again highlights the several possible interactions and synergisms between COPD and HF [12] that did worsen the overall patient prognosis. In our stratification survival analyses, patients with COPD + HF had a worse mortality rate for all causes and respiratory or cardiovascular causes (Fig. 2). The presence of dementia, a predictor of death in elderly patients [42], was independently associated with mortality at one year.

The major strengths of the present study are the large and real-life cohort of elderly patients recruited and the novelty of an approach based upon the evaluation of these distinct patient categories. As for the limitations, we should mention the retrospective nature of the analyses derived from a dataset registry, the relatively short follow-up (one year), and the lack of precise spirometric data confirming the persistent airflow limitation of COPD patients. This limitation, which on the other hand is common to most of the studies published in the field, could have induced a an overdiagnosis of COPD mainly based on clinical findings and historical informations [43]. With this caution in mind, this retrospective analysis demonstrates that hospitalized elderly patients with COPD and HF have a worse prognosis than those with COPD or HF alone. This finding may help to plan and tailor therapeutic and management interventions for these patients.

## Appendix

ICD-9 Codes

Hypertension: 40.x

Hypercholesterolemia 272.0

Coronary artery disease: 411.1, 413.x, 414.8, 414.9

Atrial fibrillation: 427.31

Peripheral artery disease 440.2.x, 440.4.x, 443.9.x

Diabetes: 250.x

Chronic kidney disease: 585.x

Osteoporosis: 733.0, 733.01

Dementia: 290.x, 294.1

Depression: 296.2, 296.3, 311.0

Cancer: 14.x, 15.x, 16.x, 17.x, 18.x, 19.x, 20.x

x: refers to all the subsequent subcodess
